# Valorization of baobab seeds (*Adansonia digitata)* as a coffee-like beverage: evaluation of roasting time on bioactive compounds

**DOI:** 10.1007/s13197-023-05873-2

**Published:** 2023-12-09

**Authors:** Etivaldo Marcolino, Diogo Salavarria, Luíz Guilherme Malaquias da Silva, Adelaide Almeida, Flávia Matias Oliveira da Silva, Carlos Ribeiro, João Dias

**Affiliations:** 1 Instituto Politécnico de Beja, Escola Superior Agrária de Beja, Rua Pedro Soares, 7800-295 Beja, Portugal; 2 Instituto Federal de Educação, Ciência e Tecnologia do Sul de Minas, Rod. Machado - Paraguaçu, S/N - Santo Antonio, Machado, MG 37750-000 Brazil; 3 GeoBioTec - Geobiosciências, Geoengenharia e Geotecnologias, Universidade Nova de Lisboa, Campus da Caparica, 2829-516 Monte da Caparica, Portugal; 4 FibEnTech - Materiais de Fibra e Tecnologias Ambientais, R. Marques de Ávila e Bolama, 6201-001 Covilhã, Portugal

**Keywords:** Antioxidant activity, Baobab, Caffeine, Total phenols, Sustainability

## Abstract

The baobab tree (*Adansonia digitata*) can be found in sub-Saharan Africa, and its fruit presents high nutritional value. However, baobab seeds are often discarded and their potential remains underutilized. This study aimed to investigate the effect of roasting time (30/55/80/105 min at 200 °C) on the physical–chemical properties of baobab seeds and the bioactive compounds in a coffee-like beverage. The results showed a decrease in moisture, Aw (water activity), and hardness of baobab seeds with increasing roasting time. These changes resulted from moisture loss, caramelization, and Maillard reactions, which also affected appearance when compared with unroasted baobab seeds. The pH of the beverage decreased to a value of around 6.01 after 105 min of roasting. The total phenolic content and antioxidant activity of the beverage increased with roasting time, reaching 851.2 mg GAE/100 g (after 80 min) and 18.9 mmol Fe^2+^/100 g (after 55 min), respectively. The caffeine content remained stable around 16 mg/100 g from 55 to 105 min, lower than that of unroasted coffee beans and decaffeinated coffee. These findings suggest the potential for valorizing baobab seeds in the development of a new coffee-like beverage with lower caffeine content.

## Introduction

Coffee is a worldwide beverage, and the consumption varies widely, depending mostly on cultural background and economical reasons. According to recent data, the world coffee consumption in 2020/2021 was 166.3 million 60-kg bags, representing an 1.3% increase considering the previous year (ICO 2023). Several factors justify this evolution, like its unique aroma and flavour, stimulant effect or high level of antioxidants (Mostafa et al. 2021), consequence of a large chemical spectrum of volatile compounds (Pinheiro et al. 2021). Such chemical composition is related with genetic factors, postharvest processing, Maillard and caramelization reactions. Caffeine (1,3,7-trimethylamine) is a methylxanthine, commonly found in some plants, like coffee (Liao et al. 2022) and cocoa (Panda et al. 2022), and its concentration depends on genetic factors, crop location or harvesting time. The quality of coffee brew, such as espresso, is influenced by coffee variety, origin, presence/type of defects, water pressure (Klotz et al. 2020) and roasting degree (Liao et al. 2022). Caffeine is easily absorbed by human organism and is associated with high antioxidant activity (Pinheiro et al. 2021). Although presents no side effects on healthy consumers, caffeine may trigger several neuroendocrine and cardiovascular effects (Grant et al. 2018). In fact, excessive consumption of coffee has been associated with negative effects such as insomnia, depression, hypertension, coronary heart disease, anxiety, and even pregnancy-related problems (Ciaramelli et al. 2019). Consequently, there is a growing interest in coffee substitutes, with similar sensorial properties but without the negative impact on health (Mostafa et al. 2021), including cereals, chicory, acorn, chickpeas, dried figs or Jerusalem artichoke. In fact, coffee substitutes not only present negligible concentrations of caffein in the obtained beverages, but also present high concentrations of bioactive compounds such as vitamins, minerals and polyphenols (Samsonowicz et al. 2019).

The baobab tree (*Adansonia digitata* L.) belongs to Malvaceae family and is mostly found in the sub-Saharan Africa, including Angola, Tanzania, Sudan (Monteiro et al. 2022), and in western Madagascar (Ismail et al. 2022). Some specimens are claimed to be over 1000 years (Rahul et al. 2015) and, seasonally, about 200 kg of fruit is produced per tree (Sidibe and Williams 2002). Even today, small rural communities acknowledge the importance of baobab as a source of food, in the formulation of medicines and livelihood (Ismail et al. 2022). In fact, baobab tree may be considered a sustainable tree as all vegetative parts are used: (1) pulp is used as food or medicine, due the presence of bioactive compounds, such as phenolic compounds (Ismail et al. 2022; Monteiro et al. 2022), ascorbic acid, minerals or vitamins (Besco et al. 2007); (2) fibers are used for making ropes; (3) bark is used for producing decorative objects or as firewood; (4) seeds are used as feedstock, in the production of flour and oil extraction for cosmetic applications (Monteiro et al. 2022). Baobab seeds are numerous and large, surrounded by a whiteish naturally dry pulp, and remaining viable over 5 years (Gebauer et al. 2016). Literature reports the high potential of baobab seeds as a functional food ingredient, due to the content of phytosterols, proteins, fibers, minerals, vitamins (A, C, E, and D3), playing a key role in cell regeneration, delaying aging (Ames 2018).

Recently, United Nations identified *responsible consumption and production* as one of the seventeen sustainable development goals in the “2030 Agenda for Sustainable Development”, pointing the need of actions to improve resource efficiency and avoid over-extraction. The contribution of *A. digitata* towards such goal cannot be neglected, and the use of seeds in the production of coffee-like beverages represents an important alternative to conventional crops with higher environmental impact. The objective of this work was the development and characterization of a coffee-like beverage, using baobab seed, and the evaluation of the impact of roasting degree in the antioxidant, phenolic and caffein content of the brew.

## Materials and methods

### Baobab seeds preparation

**Fig. 1:**
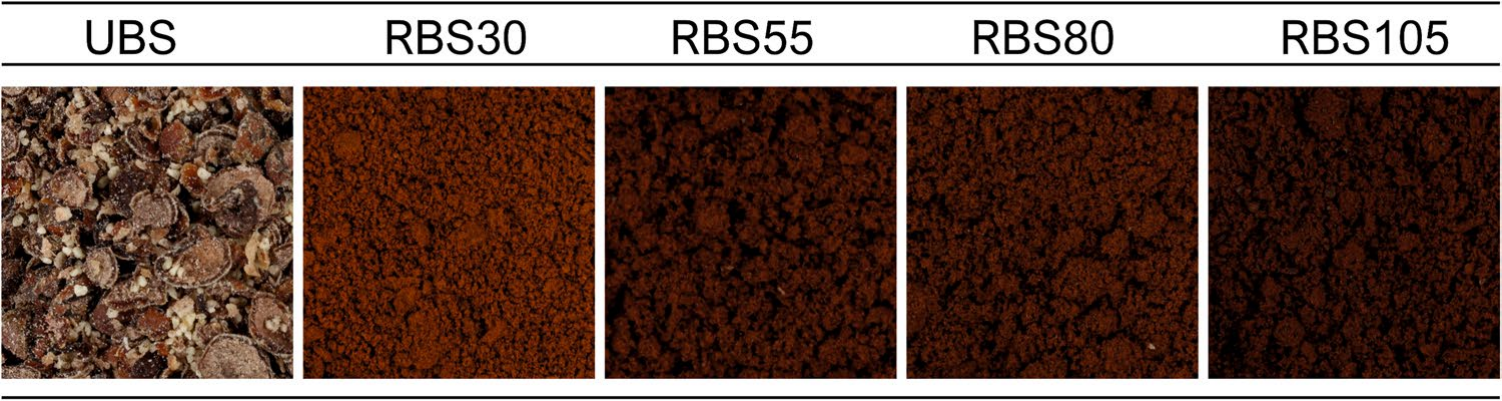
Ground samples of baobab seeds after different roasting times

Baobab pulp with seeds was obtained from a local store in Lisbon (Portugal), then hermetically sealed in polypropylene bags and transported at 20 °C to Instituto Politécnico de Beja. Then, pulp with seeds was simmered in tap water (30 °C) for the separation of seeds and washed with running water. Seeds were oven-dried overnight at 60 °C. Roasting was performed in a conventional oven (Memmert Modell 400, Germany) at 200 °C for 30 min (RBS30), 55 min (RBS55), 80 min (RBS80) and 105 min (RBS105). In addition, unroasted baobab seeds (UBS), unroasted coffee beans (UCB) and commercial decaffeinated coffee beans (DeCB) were also considered. Samples were milled using a basic knife mill and passed though a 0.5 mm sieve (Fig. 1).

### Brewed baobab preparation

Brews were prepared through infusion of ground unroasted coffee beans (UCB), decaffeinated coffee beans (DeCB) and baobab seeds with different roasting times (UBS, RBS30, RBS55, RBS80, RBS105) in distilled water for 5 min, in a solid–liquid ratio 1:20, at 99 °C using an electric plate heater (JP Selecta, Agimatic-N, Spain) and filtered with a Whatman 41 filter paper.

### Analytical method

Moisture was determined according to the gravimetric method (Monteiro et al. 2022), drying 2 g of milled samples in a oven (Memmert Modell 400, Germany) at 103 ± 1 °C to constant weight. The Aw of milled samples was measured at 20 ± 1 °C for 15 min, using a Rotronic Hygropalm HP23-A (Bassersdorf, Switzerland). Hardness was measured by compression, of individual coffee beans and baobab seeds, using a TA.XT plus 100 texture analyzer (Stable Micro Systems, United Kingdom) equipped with a 3 mm diameter aluminum probe and speed 0.2 mm/s. The color of ground baobab seeds was determined by image analysis using a Canon EOS M6 digital camera (f/16, exposure ¼, ISO100, no flash, no zoom, 6000 × 4000 pixels), based of Dias et al. (2020). The obtained RGB values were converted to CIE L*a*b* values according to (Hunt and Pointer 2011), considering the relative spectral power distribution of illuminant D65. Color variation index (∆E), hue (H*), chroma (C*) and browing index (BI) were calculated according to the following equations (Azab et al. 2022):

1 $$\Delta E = \sqrt {\left( {\Delta L^{*} } \right)^{2} + \left( {\Delta a^{*} } \right)^{2} + \left( {\Delta b^{*} } \right)^{2} }$$

2 $$H^{*} = arctan\left( {\frac{{b^{*} }}{{a^{*} }}} \right)$$

3 $$C^{*} = \sqrt {a^{*2} + b^{*2} }$$

4 $$BI = \frac{100}{{0.17}}\left( {\frac{{a^{*} + 175L^{*} }}{{5.647L^{*} + a^{*} - 3.012b^{*} }} - 0.31} \right)$$

The pH of baobab brew was evaluated with a Halo FC2022 pH meter (Hanna Instruments, USA) at 20 ± 1 °C. All above mentioned analyses were performed in quintuplicate.

### Evaluation of bioactive compounds

Coffee and baobab aqueous extracts for total phenolic content, antioxidant activity and caffeine were performed, in triplicate, according to the procedure described by Roesler et al. (2007) with some modifications. About 0.2 g of milled sample was taken into a micro-tube and 2 mL demineralized water were added. Then, the mixture was homogenized (Bioer ThermoCell Mixing Block MB 102, Germany) at 1500 rpm for 5 min at 99 °C, simulating a coffee brew, followed by centrifugation (Hettich MiKro200, Germany) at 21000 g for 15 min. The supernatant was filtered with a Whatman 41 filter to a 10 mL volumetric flask, and the volume was made up with demineralized water. Total phenolic content was evaluated by spectrophotometry using the Folin–Ciocalteau method. Into wells of a 96-well clear microplate, a volume of 20 μL of properly diluted sample extract was mixed with 100 μL of diluted Folin–Ciocalteau reagent (1:10 v/v) and 75 μL sodium carbonate solution (75 g/L) incubated for 2 h in the dark at 20 °C. After, absorbance was measured at 740 nm in a microplate reader FLUOstar Optima (BMG Labtech, Germany). Gallic acid (1.05–21.0 mg/100 mL) was used as a standard and results were expressed in mg GAE (gallic acid equivalents) per 100 g sample (Oyama and Eagle 1956). The antioxidant activity was evaluated by determination of ferric reducing antioxidant power (FRAP) assay in accordance with Ou et al. (2002). Into wells of a 96-well clear microplate, a volume of 20 μL of properly diluted sample extract was mixed with 30 μL of water and 200 μL of FRAP reagent, previously prepared mixing acetate buffer (pH 3.6), FeCl_3_ (20 mmol/L) and TPTZ (diluted in hydrochloric acid at 40 mmol/L) in a proportion 10:10:1. After, samples were incubated in the dark at 37 °C for 8 min and absorbance was measured at 595 nm in a microplate reader FLUOstar Optima (BMG Labtech, Germany). Calibration curve was prepared with ferrous sulphate solutions at concentrations ranging 0.25–2.5 mmol/L and results were expressed in mmol Fe^2+^ per 100 g sample.

The determination of caffeine was performed in an UltiMate 3000 HPLC (Thermo Scientific, USA) with mass electrospray ionization source (ESI, in positive mode), with helium as nebulizing gas were used, controlled with software *Chromeleon™ Chromatography Data System* and *Thermo Scientific™* *Xcalibur™*. Prior to the injections, samples were diluted (1:100) later filtrated with a *nylon filter* of 0.20 μm, followed by insertion in vial*s* and placement in the automatic sampler. Was used a 100 μL injection volume (partial loop) was used with a flow rate at 25 μL/min and a gradient of (A) ultrapure water with 0.1% formic acid and (B) acetonitrile. The mobile phase used was A:B (30:70) with a flow of 0.25 mL/min. A reverse-phase column C18 (100 × 2.1 mm; 2.6 μm; AccucoreaQ), at 25 °C. The quantification of caffeine in the samples was performed by chromatographic analysis in isocratic mode. The sample volume (30 μL) was selected according to the calibration line prepared for the equipment according to with adaptations. Chromatograms were obtained with retention time of 1.40 s, and quantification was performed through a calibration curve (R^2^ = 0.9976) with five standard caffeine at 0.25–1.25 mg/L. The conditions to determine the caffeine content was done according to Dordio et al. (2010) and Almeida et al. (2022), with some modifications.

Reference standard caffeine (≥ 99% purity), HPLC-grade acetonitrile and methanol from Sigma-Aldrich. Folin–Ciocalteau reagent from Sigma-Aldrich (USA). Water was prepared from a Millipore MilliQ system (Bedford, MA, USA). Formic acid (98%) was purchased from Merck. The remaining reactives were purchased from Panreac (Barcelona, Spain).

### Statistical analysis

Descriptive statistical analyses were performed with software Statistica 12 (StatSoft, USA). Data was presented as means ± standard deviation. Statistical variation among samples was determined using analysis of variance (ANOVA), followed by Tukey’s post hoc test (*P* < 0.05).

## Results and discussion

The results of moisture, Aw, hardness, color parameters and pH of the baobab samples before extraction, are expressed in Table 1.

**Table 1:** Results of moisture, Aw, hardness, color parameters and pH

	Moisture (%)	Aw (−)	Hardness (kgf)	∆E (−)	L* (−)	C* (−)	H* (−)	BI (−)	pH (−)
UBS	10.9 ± 0.2^a^	0.37 ± 0.01^a^	20.82 ± 4.62^a^		38.7 ± 3.0^a^	31.6 ± 4.3^a^	0.94 ± 0.10^a^	133.7 ± 15.2^a^	7.42 ± 0.06^a^
RBS30	3.0 ± 0.1^b^	0.28 ± 0.03^ab^	7.47 ± 1.53^b^	29.7 ± 2.1^b^	19.7 ± 1.1^b^	9.3 ± 2.2^b^	0.75 ± 0.30^a^	60.3 ± 14.2^b^	6.80 ± 0.02^b^
RBS55	1.9 ± 0.2^c^	0.19 ± 0.06^ cd^	4.01 ± 3.25^bc^	30.8 ± 0.8^b^	19.6 ± 1.3^b^	7.6 ± 0.4^bc^	0.76 ± 0.19^a^	50.0 ± 4.5^b^	6.61 ± 0.02^c^
RBS80	1.1 ± 0.1^d^	0.27 ± 0.05^bc^	3.96 ± 0.57^bc^	31.1 ± 1.4^ab^	19.3 ± 1.4^b^	7.3 ± 1.0^bc^	0.72 ± 0.04^a^	48.8 ± 6.0^b^	6.29 ± 0.02^d^
RBS105	0.2 ± 0.1^e^	0.18 ± 0.06^d^	3.11 ± 0.76^c^	33.9 ± 1.2^a^	19.3 ± 1.0^b^	3.7 ± 1.0^c^	0.87 ± 0.13^a^	24.3 ± 6.0^c^	6.01 ± 0.01^e^

*UBS* unroasted baobab seeds, *RBS30* baobab seeds roasted for 30 min, *RBS55* baobab seeds roasted for 55 min, *RBS80* baobab seeds roasted for 80 min, *RBS105* baobab seeds roasted for 105 min

Values number in the same column followed by different superscripts are significantly different at *P* < 0.05; Each value is an average of five replicates; ± Denotes standard deviation among the replicates

Moisture content of unroasted baobab seeds (UBS) was 10.9%, decreasing progressively with roasting time (Table 1), as expected. The minimum observed value was 0.2%, after 105 min (RBS105), similar to the usual moisture value on roasted coffee beans (Nogueira and Koziorowski 2020). According to Pittia et al. (2007), water vapor is formed during pyrolysis reaction, thus the variation of moisture content includes the balance of the original water content in unroasted beans and the water formed and released as a consequence of the pyrolysis. The variation of Aw with roasting time was similar to moisture, the highest value (Aw = 0.37) was observed on unroasted sample (UBS), decreasing progressively with roasting time to a minimum at 105 min (Aw = 0.18), similar to the obtained in medium and dark roasted coffee beans in previous studies (Pittia et al. 2007).

Hardness presented a progressive decrease with roasting time, to a minimum value around 3.11 kgf at 105 min. In fact, literature refers the impact of roasting process on the texture of coffee bean, becoming brittle and fragile. Such textural changes are consequence of a modification on coffee composition due to nonenzymatic browning and pyrolysis, causing a decrease on density (Pittia et al. 2007). Such textural change presented a significant impact on the grinding process of roasted beans, influencing the aqueous extraction of the beans.

The appearance of coffee, or coffee-like beverages, is a major parameter for evaluating quality due the influence on the brew and is usually used as an indicator for monitoring roasting process (Shan et al. 2016). Computer vision is a solid alternative for color evaluation and used in different sectors of food industry, such as chocolate (Dias et al. 2022), cheese (Dias et al. 2020) and also coffee (Leme et al. 2019). In this study, computer vision was used to evaluate the color of ground baobab seeds and calculate color parameters ∆E, C*, H* and BI, as presented in Table 1. Color variation index (ΔE) presented values between 29.7 and 33.9, indicating a variation in the color of roasted baobab beans, compared with UBS sample, in agreement with the visual observation (Fig. 1). Also, color parameters lightness (L*), chroma (C*) and BI decreased after 30 min roasting time, however no significant differences were observed with longer roasting times (*P* > 0.05). Such change into a darker appearance results from the formation of melanoidins, consequence of Maillard and caramelization reactions (Leme et al. 2019), but with a large impact on sensory perception.

The pH value of aqueous coffee extracts is a measurement correlated with perceived acidity and the variation depends on the degree of dissociation of organic acids such as oxalic, tartaric, malic or citric (Pinheiro et al. 2021). Unroasted baobab sample (UBS) presented a pH of 7.42, decreasing with roasting time to 6.01 (RBS105), as presented in Table 1. The impact of roasting process in the pH of baobab brews was studied before, with similar values and also decreasing with roasting degree (Ismail et al. 2022). These observations do not agree with the observed on coffee brews, where pH increases with roasting degree, consequence of the reduction on soluble protonated acidic compounds (Ismail et al. 2022).

Phenolic compounds are result of the degradation of chlorogenic acids, found in green coffee beans (Hwang et al. 2012). The determination of TPC in coffee and coffee-like beverages has been widely studied and represents a significant part of the bioactive compounds found in coffee brew, together with caffeine (Liao et al. 2022; Pinheiro et al. 2021). Such compounds present significant biological effects such as antimicrobial, cardioprotective and stimulation of nervous system (Pinheiro et al. 2021). The results of TPC of baobab brews (Table 2) presented values around 275.1 mg GAE/100 g on UBS, increasing with roasting time up to 851.2 mg GAE/100 g, after 80 min roasting time (RBS80). Such observations are consequence of the formation of phenolic compounds from the degradation of chlorogenic acids during roasting process (Pinheiro et al. 2021) and has been reported previously in coffee substitutes such as hawthorn, chicory or artichoke (Mostafa et al. 2021). Also, the TPC values of baobab brews (Table 2) were significantly lower than UCB (1601.4 mg GAE/100 g) and DeCB (1556.7 mg GAE/100 g), consequence of the lower TPC content in baobab beans (Monteiro et al. 2022; Liao et al. 2022) and similar to previous studies (Ismail et al. 2022).

**Table 2:** Results of TPC, antioxidant activity and caffeine from different brews

	TPC (mg GAE/100 g)	FRAP (mmol Fe^2+^/100 g)	Caffeine (mg/100 g)
UBS	275.1 ± 39.2^f^	8.2 ± 1.5^ h^	17.3 ± 0.1^d^
RBS30	602.0 ± 21.2^ed^	14.0 ± 0.4^efg^	21.2 ± 0.2^d^
RBS55	763.8 ± 47.6^cb^	18.9 ± 0.3^c^	16.2 ± 0.1^efgh^
RBS80	851.2 ± 17.0^b^	17.7 ± 0.9^ cd^	16.1 ± 0.1^fgh^
RBS105	732.6 ± 6.3^cb^	15.2 ± 0.2^de^	16.6 ± 0.3^efgh^
UCB	1601.4 ± 45.0^a^	26.8 ± 0.8^ab^	1139.5 ± 2.8^a^
DeCB	1556.7 ± 12.3^a^	24.3 ± 1.1^ab^	123.3 ± 0.7^b^

*UBS* unroasted baobab seeds; *RBS30* baobab seeds roasted for 30 min; *RBS55* baobab seeds roasted for 55 min; *RBS80* baobab seeds roasted for 80 min; *RBS105* baobab seeds roasted for 105 min; *UCB* unroasted coffee beans; *DeCB* commercial decaffeinated coffee beans

Values number in the same column followed by different superscripts are significantly different at *P* < 0.05; Each value is an average of three replicates; ± Denotes standard deviation among the replicates

The FRAP mechanism is usually used to evaluate the capacity of the antioxidant to stabilize free radicals, used in the evaluation of antioxidant activity of coffee and coffee-like beverages (Acidri et al. 2020). Based on available literature, the absolute values of AA depend largely on the followed methodology, nevertheless FRAP generally presents better results than ABTS assay, also known as Trolox equivalent antioxidant capacity (TEAC), or 2,2-Diphenyl-1-picrylhydrazyl (DPPH) assay. The AA results of baobab brews (Table 2) presented the initial value 8.2 mmol Fe^2+^/100 g (UBS), increasing until 55 min roasting time (18.9 mmol Fe^2+^/100 g) and decreasing later until 105 min (15.2 mmol Fe^2+^/100 g), similar to the reported previously concerning TPC (Table 2). As observed in Table 2, AA results of baobab brews were significantly lower than UCB (26.8 mmol Fe^2+^/100 g) and DeCB (24.3 mmol Fe^2+^/100 g). Previous reports also observed a correlation between TPC and AA on coffee extracts (Bobkova et al. 2020) and coffee substitutes (Samsonowicz et al. 2019). In fact, chlorogenic acid and polyphenols such as caffeic, ferulic and *n*-coumaric acids are identified as some of the compounds related to the antioxidant properties of coffee (Pinheiro et al. 2021; Bobkova et al. 2020). The impact of roasting degree in AA has been reported in previous studies (Alamri et al. 2022; Acidri et al. 2020), however results are not conclusive and may be dependent on processing conditions (Ismail et al. 2022).

Caffeine is an alkaloid found in tea and coffee, odorless, with pronounced bitterness and important in the development of the flavour of brews (Ismail et al. 2022; Pinheiro et al. 2021). Actually, it is the most consumed psychoactive substance throughout the world, identified as stimulant of the central nervous system (Masi et al. 2016), with beneficial effects on neurological diseases and decreasing the risk of type 2 diabetes. Despite these positive impacts, caffeine intake is also related with nervousness, anxiety or increase on blood pressure (Belguidoum et al. 2014). In the present study, caffein of baobab brews (Table 2) presented results around 17.3 mg/100 g, for UBS sample, significantly lower than UCB (1139.5 mg/100 g) and DeCB (123.3 mg/100 g), consequence of the lower caffein content of baobab seeds (Olaitan et al. 2014). Additionally, caffeine increased from 17.3 mg/100 g (UBS) to 21.2 mg/100 g (RBS30) during the early 30 min roasting time, consequence of the drying effect (Table 1). However, from 55 to 105 min caffeine values did not present significant differences (*P* > 0.05), indicating a significant heat stability, as observed in previous reports (Alamri et al. 2022).

## Conclusions

The current study focused on the evaluation of roasting time on the physico-chemical properties of baobab seeds and bioactive compounds of baobab brew, compared to unroasted coffee beans and decaffeinated. The effect of roasting time on baobab seeds was similar to the reported on coffee beans, namely lower water content, lower hardness and darker appearance, consequence of pyrolysis, caramelization and Maillard reactions. The results showed a decrease on the pH of baobab brew with roasting time, contrary to the reported for coffee beans. A correlation was observed between TPC and AA, with similar evolution during roasting time and obtaining maximum values around 55–80 min. Caffeine presented a solid heat stability, with values significantly lower than decaffeinated, making roasted baobab seeds a valid option for consumers with caffeine sensitivity.

## Data Availability

All data is available within the manuscript.
